# Neoadjuvant Pembrolizumab Combined with Chemotherapy for Locally Advanced Resectable Oral and Oropharyngeal Squamous Cell Carcinomas: A Single-center Retrospective Study

**DOI:** 10.7150/jca.122201

**Published:** 2026-01-01

**Authors:** Xinrong Geng, Yuanyuan Zhao, Yunteng Wu, Xuhui Ma, Ronghui Xia, Xuanli Xu, Guoxin Ren

**Affiliations:** 1 Department of Oral Maxillofacial-Head and Neck Oncology, Shanghai Ninth People's Hospital, Shanghai Jiao Tong University School of Medicine, Shanghai, China.; 2 National Clinical Research Center of Stomatology, Shanghai, China.; 3 Shanghai Key Laboratory of Stomatology & Shanghai Research Institute of Stomatology; National Clinical Research Center of Stomatology, Shanghai, China.; 4 Department of Pathology, Shanghai Ninth People's Hospital, Shanghai Jiao Tong University School of Medicine, Shanghai, China.; 5 School of Stomatology, Shandong Second Medical University, China.

**Keywords:** locally advanced resectable oral and oropharyngeal squamous cell carcinoma, neoadjuvant, immunotherapy, pembrolizumab

## Abstract

**Objective:** Neoadjuvant chemotherapy regimens have shown encouraging efficacy characterized by high objective response rate (ORR), pathologic complete response (pCR) rate, and major pathologic response (MPR) rate, alongside acceptable safety. This single-center retrospective study aimed to evaluate the safety and efficacy of neoadjuvant pembrolizumab plus chemotherapy in patients with locally advanced resectable oral and oropharyngeal squamous cell carcinomas (LA-OSCC/OPSCC).

**Materials and methods:** A total of 50 patients were included. The patients received 2-4 cycles of neoadjuvant therapy with pembrolizumab, albumin-bound paclitaxel and cisplatin before surgery, followed by adjuvant radiotherapy or immunotherapy.

**Results:** The median follow-up time was 31.7 months (95%CI, 29.4-34.0). The ORR was 85.4%, and the MPR rate was 65.8%. The 1-year event-free survival (EFS) rate was 88.8% (95%CI, 79.8%-98.8%). Patients with moderate programmed cell death ligand 1 (PD-L1) expression (combined positive score (CPS) 1 to <10) achieved the highest MPR rate (71.4%), underscoring the potential predictive value of PD-L1 expression. Treatment-related adverse events (TRAEs), most commonly alopecia, anemia, neutropenia, and nausea, were manageable. No treatment-related deaths occurred.

**Conclusion:** This retrospective analysis indicates that neoadjuvant pembrolizumab combined with chemotherapy is a promising strategy for patients with LA-OSCC/OPSCC. Future prospective studies with larger cohorts and longer follow-up are warranted to confirm these findings.

## 1. Introduction

Oral and oropharyngeal squamous cell carcinoma (OSCC/OPSCC) ranks as the sixth most common epithelial malignancy worldwide^1^. For patients with locally advanced resectable oral and oropharyngeal squamous cell carcinomas (LA-OSCC/OPSCC), surgery resection followed by adjuvant radiotherapy or chemoradiotherapy remains the standard of care^2^. Despite multimodal treatment that includes platinum-based chemoradiotherapy, more than 50% of patients develop disease recurrence, or distant metastases within 3 years^3^,^4^,^5^. Human papillomavirus (HPV)-positive and HPV-negative oropharyngeal squamous cell carcinoma (OPSCC) constitute distinct entities with divergent molecular profiles, clinical behaviors, and therapeutic responses. HPV-negative OPSCC, which accounts for approximately 75% of all OPSCC cases, is generally associated with a poor prognosis^6^. Although HPV-positive OPSCC is recognized as a separate clinical entity with a more favorable overall prognosis, distant metastasis remains a significant therapeutic challenge, often occurring in advanced stages^7^.

Anti-programmed cell death protein 1 (PD-1) immunotherapy functions by augmenting the host antitumor immunity and inhibiting tumor cell proliferation^8^. The combination of chemotherapy and immune checkpoint inhibitors (ICIs) demonstrates efficacy by modulating the tumor microenvironment, promoting antigen release, achieving rapid induction of tumor regression, and reducing immunosuppression^9^. Immune checkpoint inhibitors (ICIs) have shown efficacy in OSCC/OPSCC, with the anti-PD-1 monoclonal antibody pembrolizumab demonstrating a particularly favorable safety profile 10.

The KEYNOTE-048 trial demonstrated that, compared to the EXTREME regimen (cetuximab plus cisplatin/5-fluorouracil), pembrolizumab combined with the PF regimen (cisplatin/5-fluorouracil) improved overall survival (OS) in patients with recurrent or metastatic head and neck squamous cell carcinoma (R/M HNSCC). Based on these results, pembrolizumab combined with chemotherapy is an established first-line treatment for R/M HNSCC, while pembrolizumab monotherapy represents a key first-line treatment for patients with programmed cell death ligand 1-positive (PD-L1+) disease 11. The combined positive score (CPS), a standardized measure of PD-L1 expression, is calculated as the number of PD-L1-positive tumor cells and associated immune cells divided by the total number of viable tumor cells, multiplied by 100. CPS has been validated as a predictive biomarker for response to pembrolizumab in HNSCC, with PD-L1 positive patients deriving greater clinical benefit from immunotherapy^12^.

The success of ICIs in R/M HNSCC has generated significant interest in the neoadjuvant application. Neoadjuvant chemoimmunotherapy for potentially resectable LA-OSCC/OPSCC aims to induce substantial pathological responses, enhance systemic immunity to prevent metastasis and recurrence, and promote tumor shrinkage. This approach may induce tumors downgrading, potentially enabling more conservative surgical interventions. Recent studies on neoadjuvant chemotherapy have demonstrated promising results, including high objective response rates (ORRs), pathologic complete response (pCR) rates, and major pathologic response (MPR) rates, alongside acceptable safety 13. The randomized, open-label, phase III clinical trial (KEYNOTE-689) evaluated the efficacy and safety of pembrolizumab as a neoadjuvant therapy, followed by standard adjuvant radiotherapy (with or without cisplatin), in patients with resectable stage III or IVA locally advanced head and neck squamous cell carcinoma (LA-HNSCC) compared with adjuvant radiotherapy (with or without cisplatin) alone. The trial has shown that neoadjuvant immunotherapy combined with postoperative immunotherapy and standard radiotherapy (with or without cisplatin) significantly improves event-free survival (EFS) and is expected to improve OS. Furthermore, successful tumor downstaging through neoadjuvant immunotherapy could potentially allow for less intensive adjuvant regimens, which might reduce the incidence of radiotherapy-related complications and directly improve patient quality of life. Consequently, this retrospective study was conducted to evaluate the safety and efficacy of neoadjuvant pembrolizumab plus chemotherapy in patients with resectable LA-OSCC/OPSCC.

## 2. Materials and Methods

### 2.1 Patient eligibility

Patients were enrolled in this retrospective study between August 2022 and December 2024. The inclusion criteria were: (a) age 18-75 years with pathologically confirmed squamous cell carcinoma; (b)stage III-IVB disease for non-oropharyngeal cancer and HPV-negative oropharyngeal cancer or stage II-III disease for HPV-positive oropharyngeal cancer according to the 8th edition of American Joint Committee on Cancer (AJCC) staging manual; (c) resectable tumor assessed by a head and neck surgeon before enrollment; (d) receipt of 2-4 cycles neoadjuvant pembrolizumab plus chemotherapy; and (e) an Eastern Cooperative Oncology Group (ECOG) performance status score of 0-1. The exclusion criteria included: (a) history of prior tumors; (b) any previous treatment; (c) presence of distant metastasis; (d) previous treatment with CTLA-4, PD-1, or PD-L1 antibodies; (e) active pulmonary disease (e.g., asthma, chronic obstructive pulmonary disease, interstitial lung pneumonia) or history of active tuberculosis; (f) active autoimmune diseases; or (g) severe hepatic or renal dysfunction.

### 2.2 Treatment

During neoadjuvant therapy, patients received 2-4 cycles of pembrolizumab (200 mg), albumin-bound paclitaxel (260 mg/m²), and cisplatin (75 mg/m²) every 3 weeks. Pembrolizumab was administered as a fixed intravenous dose of 200 mg. Albumin-bound paclitaxel and cisplatin doses were calculated based on body surface area, and were administered sequentially, with immunotherapy administered before chemotherapy. Adequate hydration was required before and after cisplatin administration for nephroprotection. All patients received prophylactic hepatoprotective and gastroprotective agents, as well as antiemetics before each cycle of therapy. Treatment modifications were necessary in two patients: one with venous thrombosis preventing deep venous access was switched to pembrolizumab plus cetuximab, and another patient transitioned to pembrolizumab monotherapy after one cycle of chemoimmunotherapy due to myelosuppression. Surgery was performed after efficacy evaluation by head and neck surgeons, typically within 4-6 weeks after immunotherapy and no later than 8 weeks. Patients subsequently received adjuvant radiotherapy according to standard protocols, initiating treatment within 6 to 8 weeks after surgery. Radiation doses were prescribed based on postoperative pathological and imaging findings. The high-risk area was typically irradiated to 60 Gy in 30 fractions for cases with negative margin and no extranodal extension, with an escalated to 66 Gy in 33 fractions for positive margins or extranodal extension, while the low-risk area received 54 Gy in 30 fractions. Patients were closely monitored throughout radiotherapy, and treatment response was assessed approximately 20 times through the treatment course. Notably, some patients who exhibited favorable responses after neoadjuvant chemoimmunotherapy were recommended to continue adjuvant pembrolizumab monotherapy postoperatively (200 mg intravenously every 3 weeks) for up to 2 years instead of adjuvant radiotherapy.

### 2.3 Endpoints

The primary endpoints were the major pathologic response (MPR) rate. Secondary endpoints included EFS, OS, ORR and safety. The MPR rate was defined as the proportion of patients with major pathological responses in tumor tissue on pathologic examination after surgical resection. The ORR, an indicator of tumor treatment efficacy, was defined as the proportion of patients achieving tumor remission within a specified period after treatment. EFS was defined as the time from randomization to the first occurrence of disease progression without surgical treatment, local or distant recurrence or death from any cause. The OS was defined as the time from randomization to death of any cause. Sample size was calculated using GraphPad Prism version 10.2.3.

## 3. Results

### 3.1 Patient clinical characteristics

**Figure 1:**
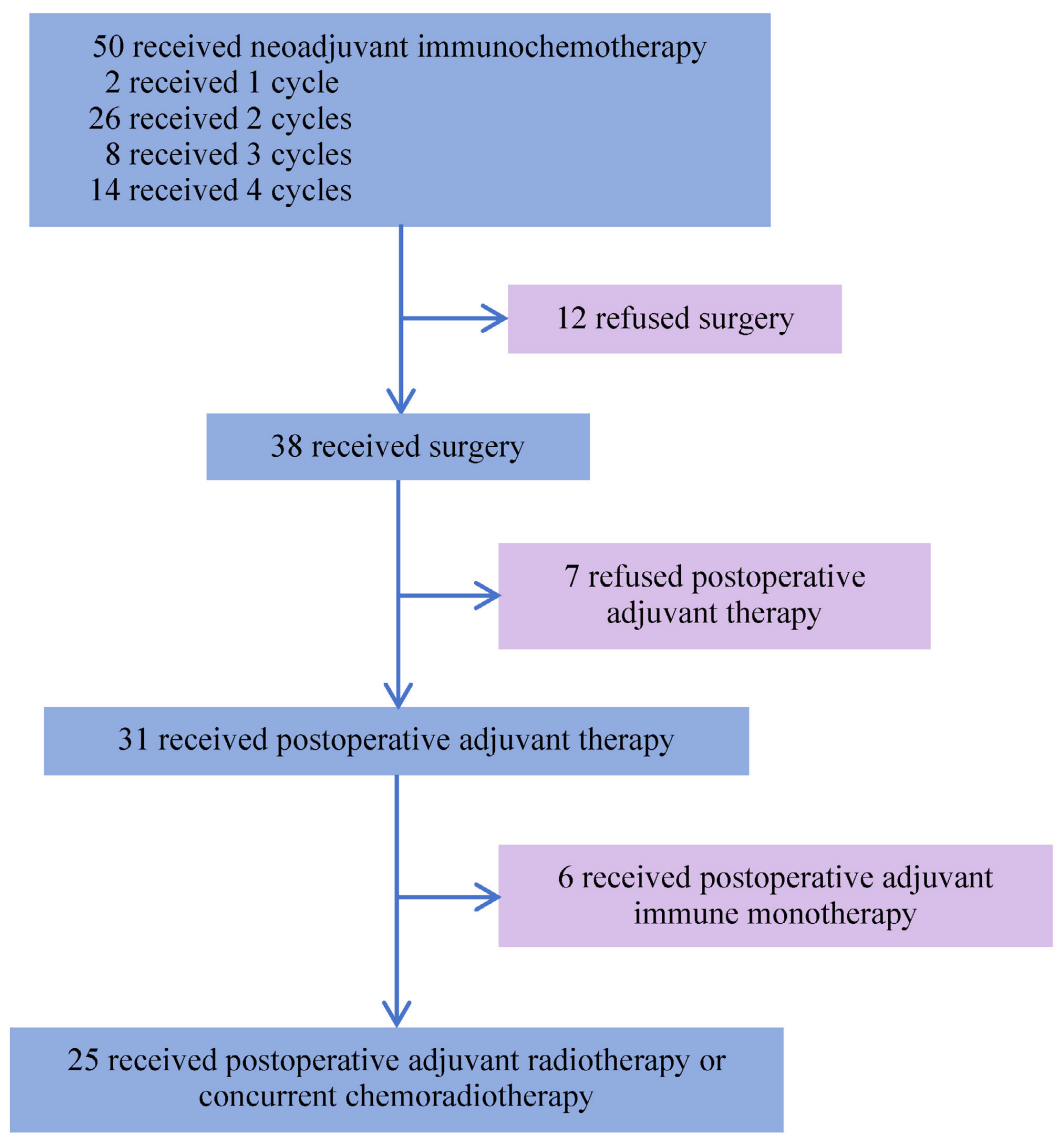
Study flowchart.

From August 19, 2022, to December 16, 2024, 50 patients with LA-HNSCC were enrolled. Their demographic and clinical characteristics are presented in **Table 1**. The median age of the patients was 58 years, 38 (76.0%) were male and 12 (24.0%) were female. Additionally, 16 (32.0%) patients had a history of smoking, and 19 (38.0%) were alcohol consumers. Among the 13 patients with oropharyngeal cancer, 6 (46.2%) were HPV-positive and 5 (38.5%) were HPV-negative, as determined by p16 IHC. PD-L1 expression was assessed in 30 patients before neoadjuvant immunotherapy, including 19 with a CPS ≥ 10, 10 with a CPS of 1 to <10, and 1 with a CPS < 1. The median follow-up duration was 31.7 months (95% CI, 29.4-34.0), defined as the interval from study enrollment to the cutoff date or death (cutoff date: June 5, 2025). All 50 patients completed neoadjuvant chemoimmunotherapy. Among them, 25 (50%) underwent surgery after 2-4 cycles, followed by adjuvant radiotherapy or concurrent chemoradiotherapy 4 weeks postoperatively. Notably, 6 (12%) patients received 2-4 cycles of chemoimmunotherapy, followed by surgical treatment, and postoperative adjuvant immunotherapy for 2 years. Furthermore, 7 (14%) patients did not receive any postoperative adjuvant therapy and 12 (24%) patients did not undergo surgery after neoadjuvant treatment **(Figure 1)**. Among the 50 patients, 47 (94%) were alive. Of the three deaths, one was attributed to surgical complications, and another died during the COVID-19 pandemic. Additionally, 47 (94%) remained recurrence-free during follow-up, while 3 patients experienced cervical lymph node metastasis.

### 3.2 Efficacy and clinical outcomes

**Figure 2:**
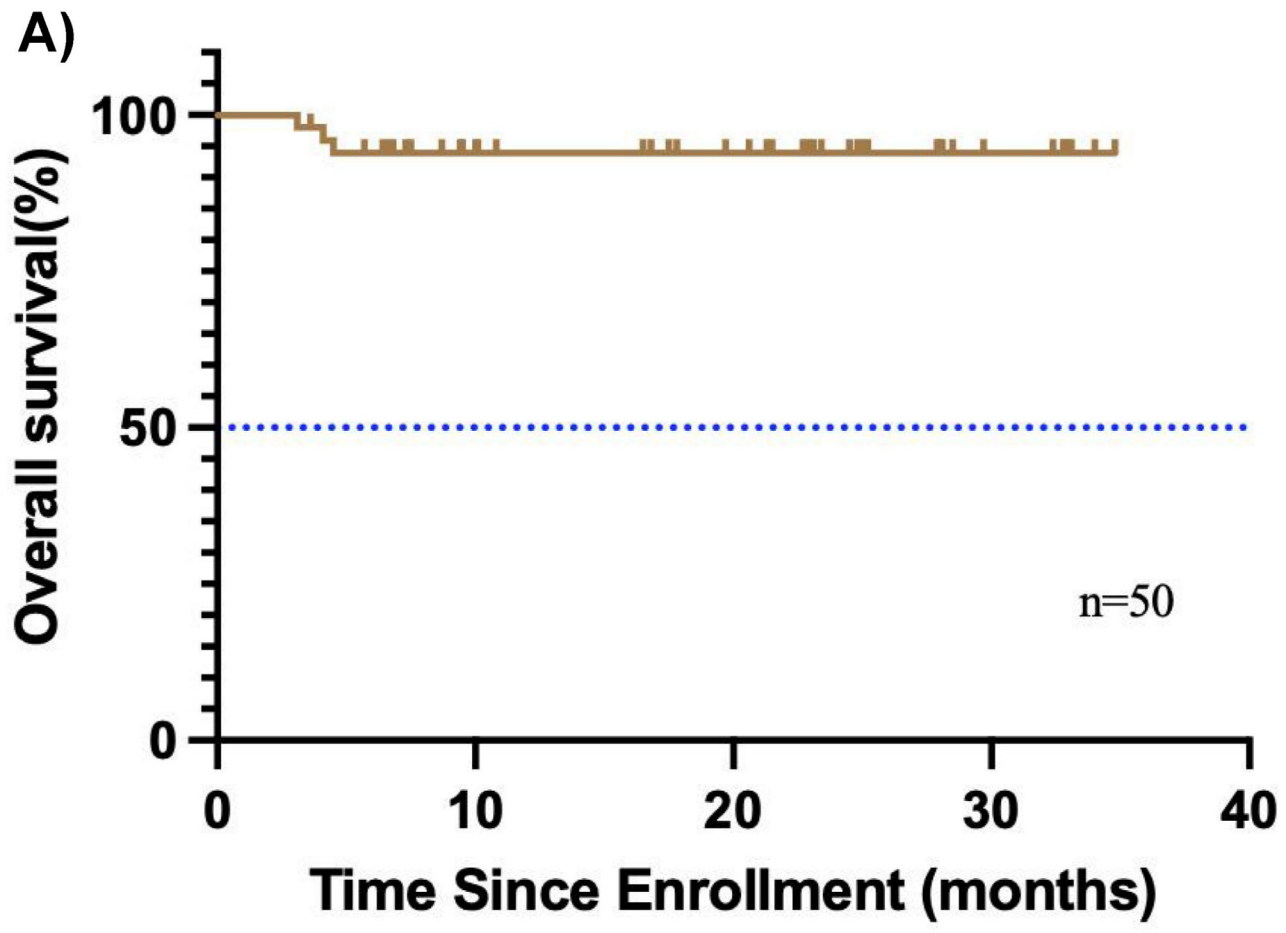

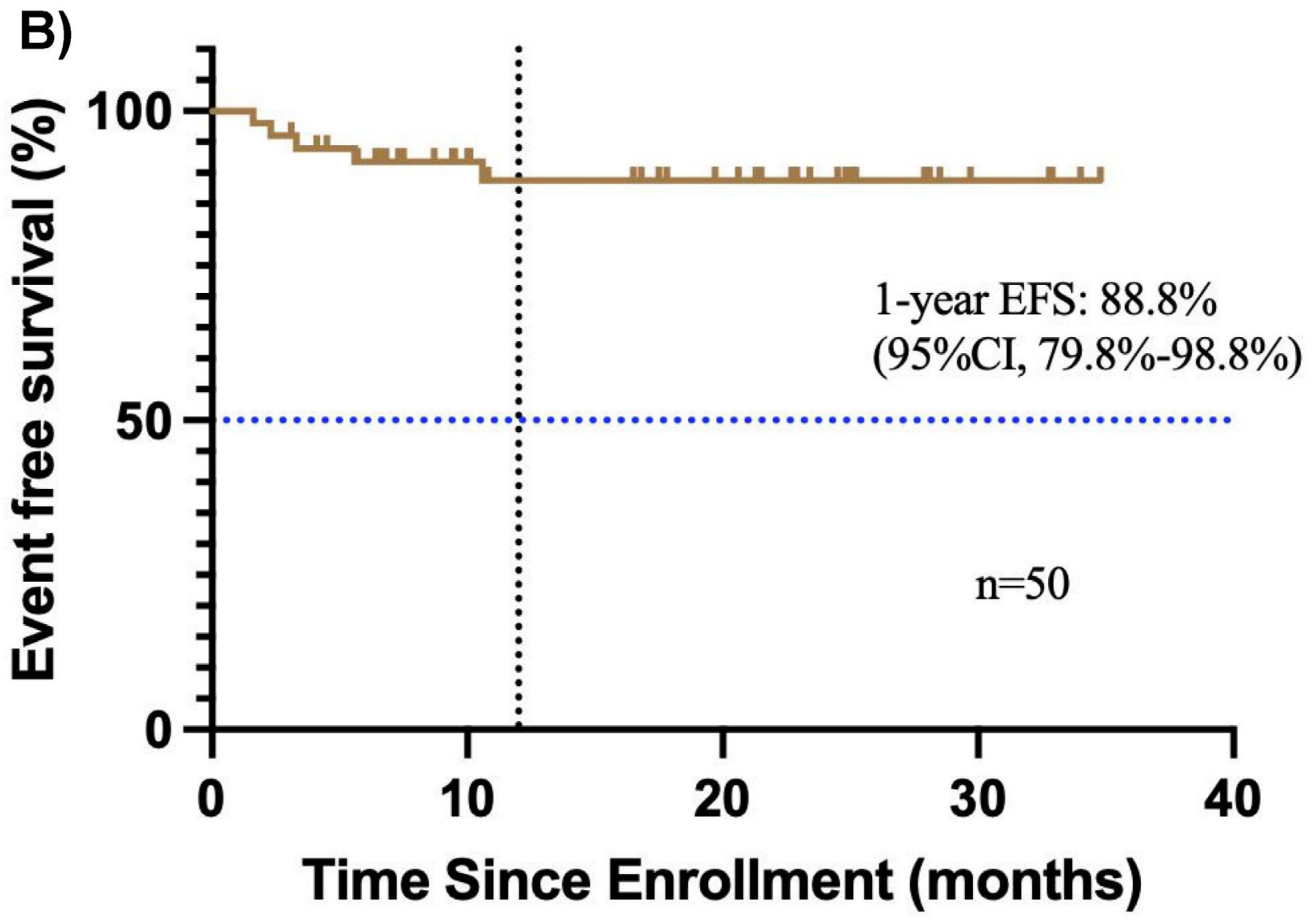
** Kaplan-Meier curves of overall survival, and disease-free survival.** (A) overall survival and (B) disease-free survival were assessed in the intention-to-treat population (n=50).

**Figure 3:**
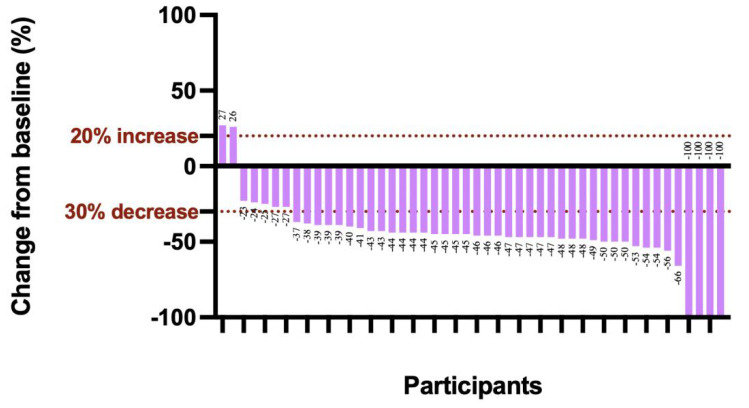
** Best percentage change from baseline in target lesion.** The dashed line at -30% change represents the RECIST version 1.1 cutoff to define partial response or complete response, and at +20% change represents the RECIST version 1.1 cutoff to define progressive disease.

The median follow-up duration was 31.7 months (95% CI, 29.4-34.0), and the median OS was not reached **(Figure 2A)**. Additionally, 3 relapses (6%) experienced relapse, all as cervical lymph node metastases. The median EFS was also not reached **(Figure 2B)**. The 1-year EFS rate was 88.8% (95% CI, 79.8%-98.8%). After neoadjuvant chemoimmunotherapy, 4 of 48 patients (22 did not undergo maxillofacial computed tomography (CT) after therapy) achieved complete response (CR), 37 achieved partial response (PR), 5 had stable disease (SD), and 2 had progressive disease (PD) **(Figure 3)**. The ORR was 85.4% (41/48), while the disease control rate (DCR) was 95.8% (46/48). Among the 50 patients, 38 underwent surgery after neoadjuvant chemoimmunotherapy, all of whom achieved an R0 resection (100%). Of these, 25 achieved MPR, resulting in an MPR rate of 65.8%.

### 3.3 Subgroup analysis

In this present study, 25 (50%) patients underwent surgery received adjuvant radiotherapy or concurrent chemoradiotherapy, while 6 (12%) received adjuvant immunotherapy as postoperative treatment. The median OS and EFS were not reached.

**Figure 4:**
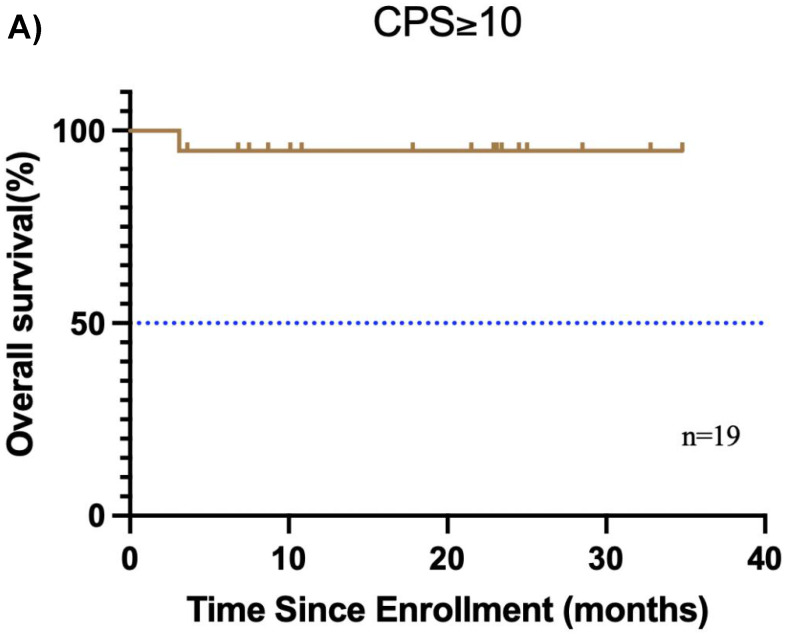

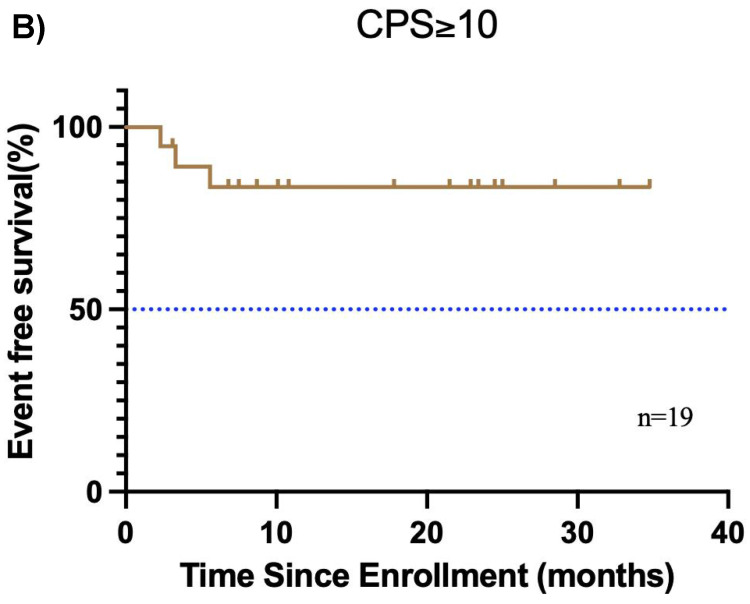

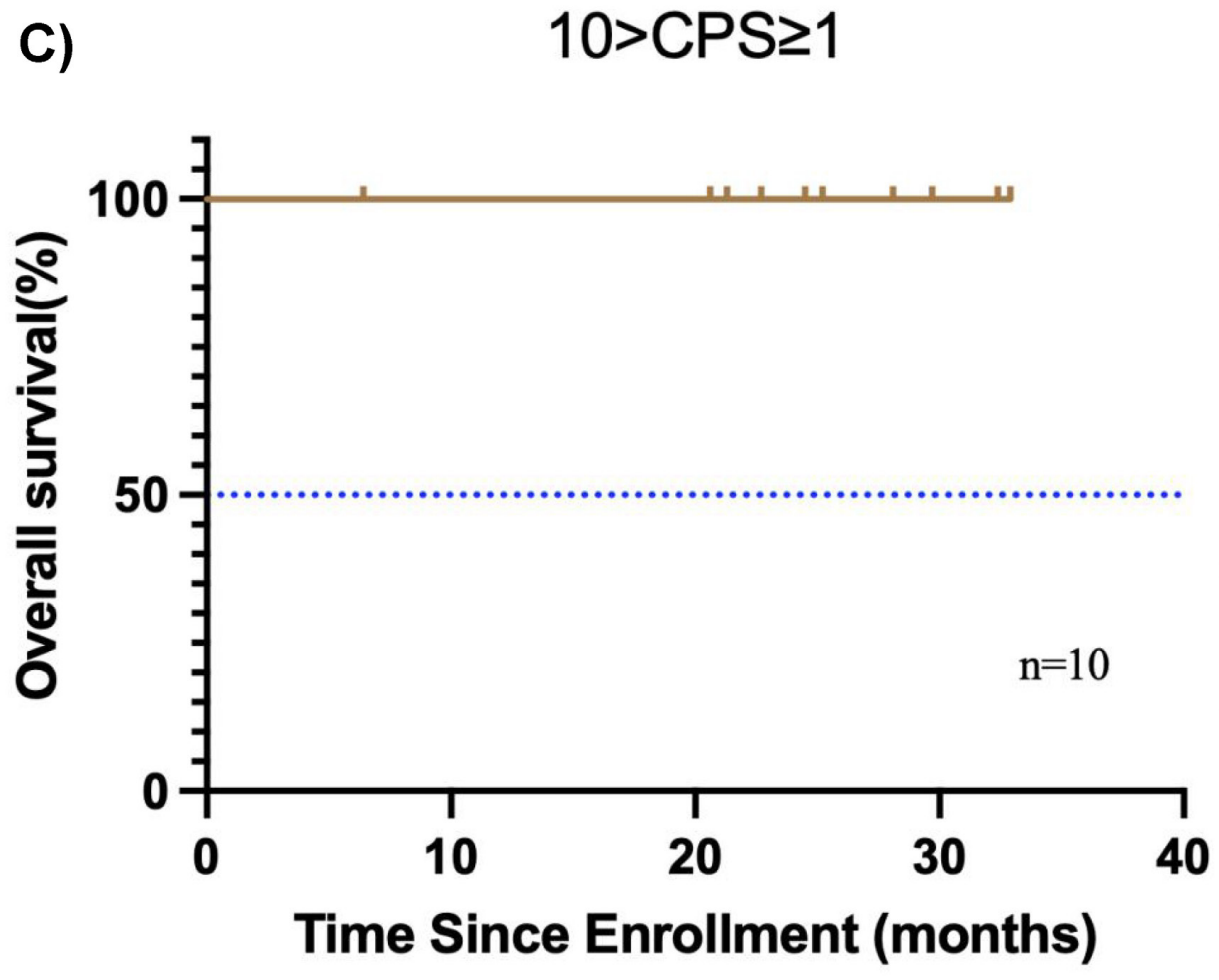

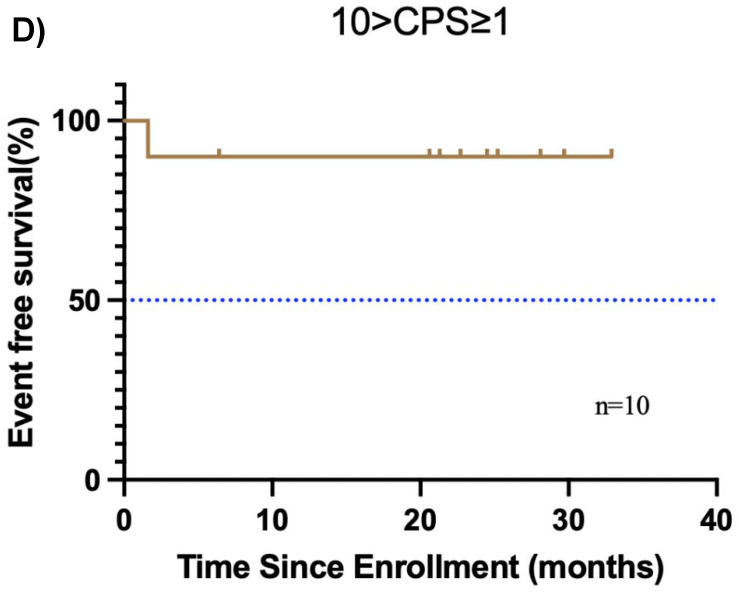

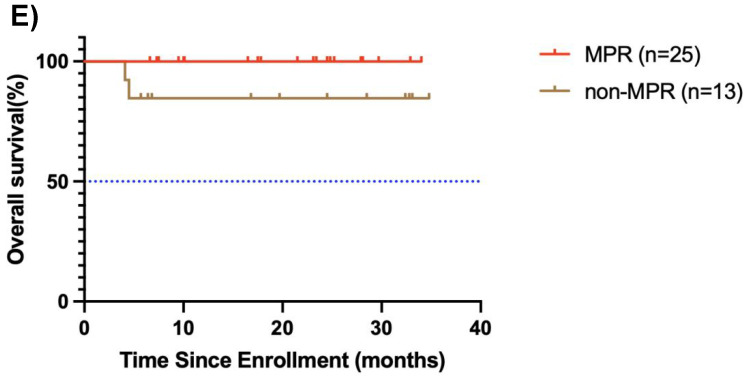

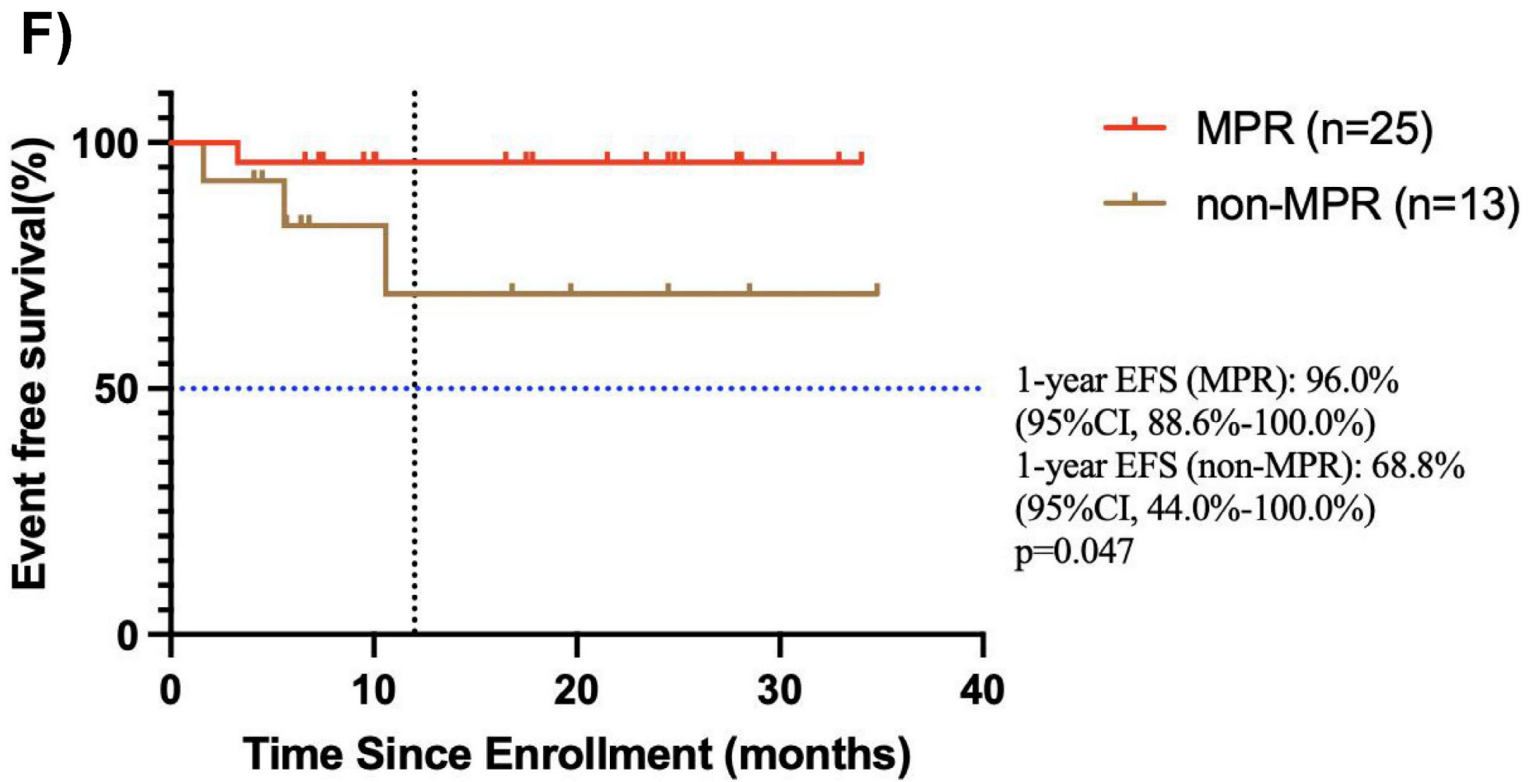
** Subgroup analysis of survival outcomes.** (A) overall survival and (B) event-free survival were assessed in the PDL1 CPS≥10 population (n=19); (C) overall survival, and (D) event-free survival were assessed in the PDL1 1 < CPS ≤ 10 population (n=10); (E) overall survival and (F) event-free survival were assessed in patients with MPR (n=25) and non-MPR (n=13).

Furthermore, the CPS was assessed in 30 patients before neoadjuvant chemoimmunotherapy. Of these, 19 (38.0%) had a CPS ≥ 10, 10 (20.0%) had a CPS of 1 to < 10, and 1 (2.0%) had a CPS < 1. In the CPS ≥ 10 group, the median OS and EFS were not reached **(Figure 4A, B)**. Additionally, 1 patient achieved CR, 14 patients achieved PR, 3 patients had SD, and 1 patient had PD, resulting in an ORR of 78.9%. Of these 19 patients, 11 underwent surgery, and 6 achieved MPR, resulting in a MPR rate of 54.5%. In the CPS of 1 to < 10 group, the median OS and EFS were not reached **(Figure 4C, D)**. 2 patients achieved CR, 5 patients achieved PR, and 1 patient had PD, resulting in an ORR of 87.5%. Notably, 2 patients did not undergo maxillofacial CT after neoadjuvant chemoimmunotherapy. Of the 10 patients, 7 patients underwent surgery, and 5 patients achieved MPR, resulting in a MPR rate of 71.4%. In the CPS < 1 group, 1 patient achieved PR, and MPR was not reached.

Among the 38 patients who underwent surgery after neoadjuvant chemoimmunotherapy, 25 achieved MPR, and the median OS and EFS were not reached. The 1-year EFS rate was 96.0 % (95% CI, 88.6%-100.0%). Of these, 3 patients achieved CR, 21 patients achieved PR, and 1 had SD, resulting in an ORR of 96%. Notably, 13 patients did not achieve MPR, and the median OS and EFS were not reached. The 1-year EFS rate was 68.8 % (95% CI, 44.0%-100.0%). In this group, 9 achieved PR, 3 had SD, and 1 had PD, yielding an ORR of 69.2% **(Figure 4E, F)**.

Among the 4 patients with CR, 3 underwent surgery, and all 3 achieved MPR (MPR rate: 100%). The median OS and EFS were not reached. Among the 37 patients with PR, 30 underwent surgery, and 21(70%) achieved MPR. The median OS and EFS were not reached. The MPR rates in patients with SD and PD were 25% and 0%, respectively, and the median OS and EFS were not reached **(Table 2)**. In the 6 cases of HPV-positive oropharyngeal carcinoma, the ORR was 66.7%. The MPR rate was 50% (2/4, 2 patients did not undergo surgery), including 1 pathologic complete response. Among the 5 HPV-negative patients, the ORR was 80%, and the MPR rate was 33.3 % (1/3, 2 patients did not undergo surgery). All HPV-positive cases exhibited high PD-L1 expression in tumor tissue (CPS ≥ 10). This finding aligns with established research indicating that HPV-related tumor microenvironments typically induce stronger PD-L1 upregulation, potentially due to the adaptive immune resistance from persistent viral antigen stimulation^14^. This analysis suggests that the favorable efficacy trend in HPV-positive patients may relate to their preexisting active tumor immune microenvironment. However, given the small sample size, these data are insufficient for definitive conclusions, and require validation in larger cohorts.

### 3.4 Safety and feasibility of neoadjuvant chemoimmunotherapy

The most frequent chemotherapy-related adverse events **(Table 3)** were alopecia (98.0%), nausea (64.0%), anemia (62.0%), neutropenia (36.0%), constipation (36.0%), elevated alkaline phosphatase (34.0%), vomiting (32.0%), fatigue (30.0%), and stomatitis (30.0%). The most common grade 3 or higher chemotherapy-related adverse events were anemia (14.0%), alopecia (8.0%), neutropenia (6.0%), thrombocytopenia (6.0%), and pyrexia (4.0%). The most frequent immune-related adverse events (irAEs) were hypothyroidism (16.0%), immune-mediated rash (10.0%), hyperthyroidism (4.0%) and immune-mediated pneumonia (4.0%). No treatment-related deaths occurred. However, 3 (6.0%) patients experienced delayed surgery due to chemotherapy-related toxicities. Additionally, 2 (4.0%) patients discontinued chemotherapy due to toxicity. One patient, unable to undergo deep vein catheterization because of venous thrombosis, was switched to immune-targeted therapy, while the other transitioned to single-agent pembrolizumab after one cycle of chemoimmunotherapy due to myelosuppression.

## 4. Discussion

**Figure 5:**
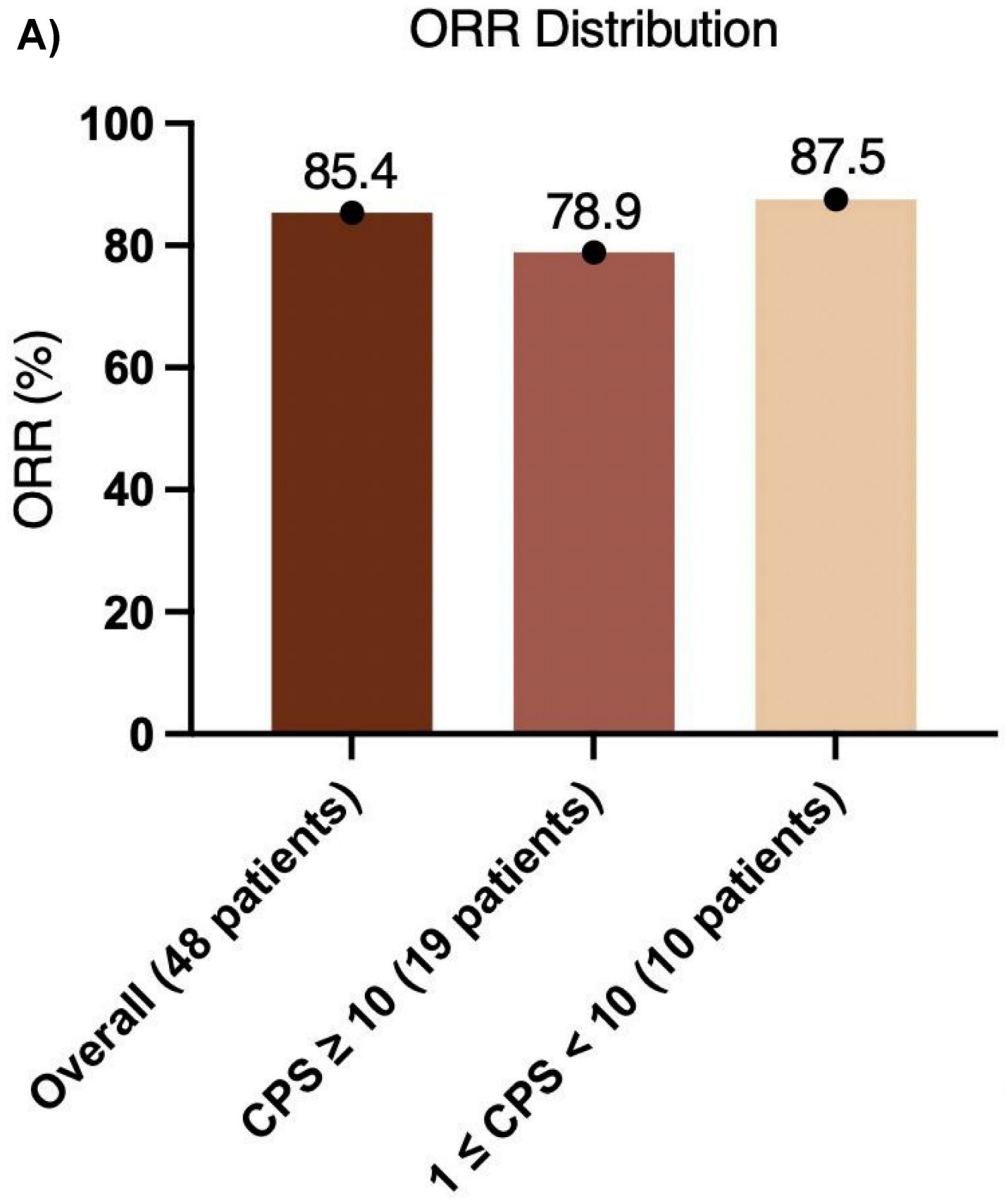

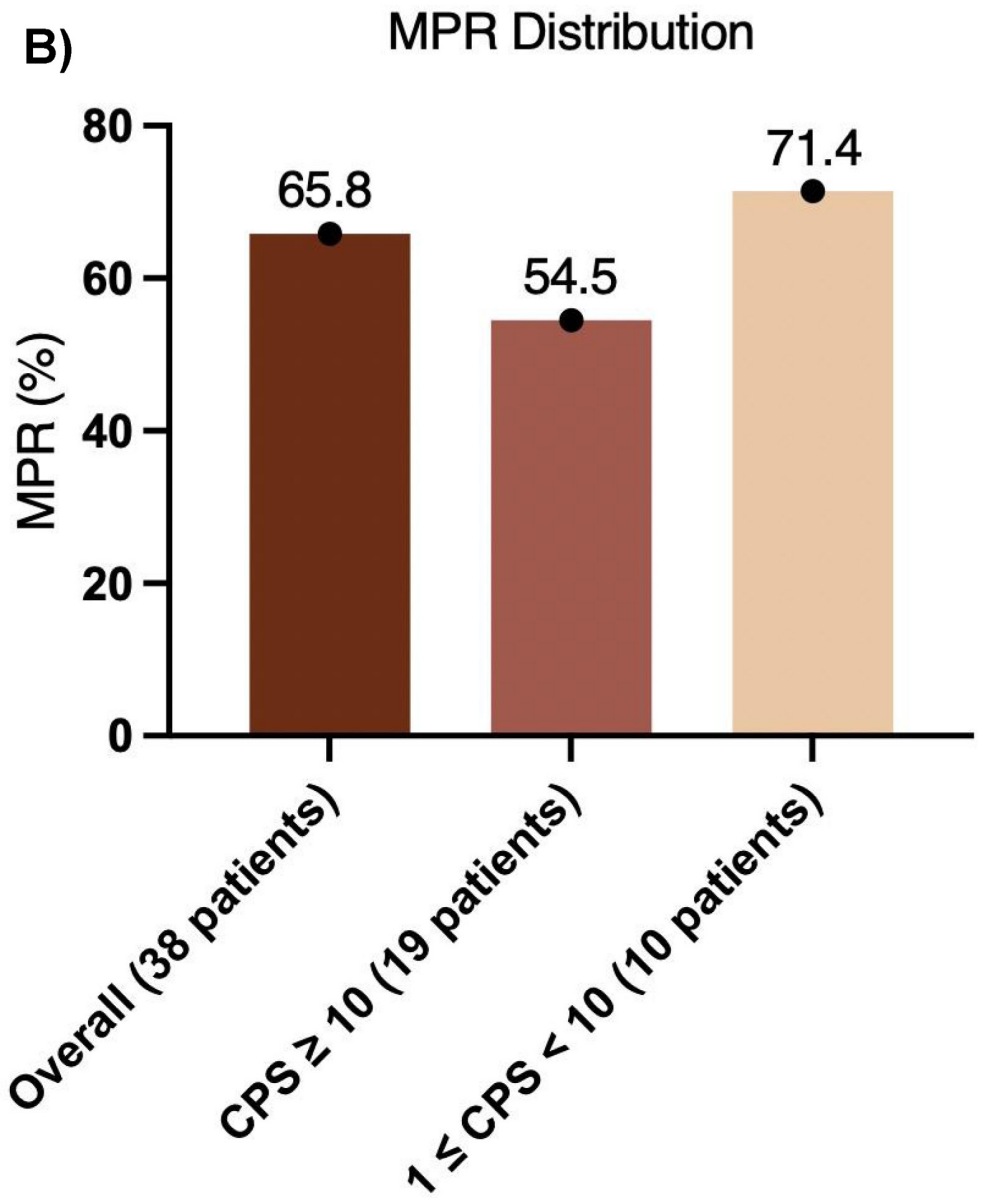
** (A) ORR distribution and (B) MPR distribution**.

Previous studies have confirmed the tolerability and potential efficacy of immunotherapy plus TP regimen chemotherapy in neoadjuvant settings for locally advanced resectable OSCC/OPSCC. The present study further supports these findings, demonstrating improved MPR and ORR. Among 48 patients who received neoadjuvant chemoimmunotherapy (2 of 50 did not undergo maxillofacial CT after treatment), the ORR was 85.4%, indicating high efficacy. This elevated ORR may also reflect the higher response rate (67.3%-86.5%) to neoadjuvant or induction chemotherapy alone in East Asian patients with OSCC/OPSCC. The ORR was 78.9% in patients with PD-L1 CPS ≥ 10 ((n = 19), and 87.5% in those with CPS of 1 to < 10 (n = 10), suggesting that moderate PD-L1 expression is associated with superior response **(Figure 5A)**. These results indicate that PD-L1 expression levels influence ORR, with moderate expression (CPS of 1 to < 10) linked to greater efficacy. Overall, the high ORR underscores the potential of this regimen for locally advanced resectable OSCC/OPSCC and the broad effectiveness of neoadjuvant chemoimmunotherapy.

Xu et al. performed a comparative meta-analysis evaluating the efficacy and safety of five chemoimmunotherapy regimens versus conventional chemotherapy. The analysis encompassed 1,856 patients with R/M HNSCC. The meta-analysis showed that, compared with standard platinum-based chemotherapy, chemoimmunotherapy significantly improved OS, PFS, and ORR, without increasing the overall incidence of adverse events, although the incidence of grade 3-4 adverse events was higher^15^.

Pathological efficacy, including MPR, represents a key endpoint in neoadjuvant therapy for OSCC/OPSCC^16^. Among the 38 patients who underwent surgery, the MPR rate was 65.8%, indicating that more than 60% achieved significant tumor pathological remission after neoadjuvant chemoimmunotherapy. This finding underscores the potential effectiveness of this regimen in locally advanced, resectable OSCC/OPSCC. Notably, pCR or major response is a recognized prognostic factor for survival. The survival benefit observed in patients with pCR may originate either from the therapeutic effect of chemotherapy or from the inherently favorable prognosis of responders. Previous research has shown that preoperative pembrolizumab combined with chemotherapy in resectable locally advanced HNSCC can yield a pCR rate of 36.4% and a MPR rate of 54.5%, without compromising surgical safety. However, in the KEYNOTE-689 study, the MPR rates in the overall immunotherapy group, the PD-L1 CPS ≥ 1 subgroup, and the CPS ≥ 10 subgroup were 9.4%, 9.8% and 13.7% respectively, all notably lower than those in our study. This discrepancy may be attributable to the use of immune monotherapy without concurrent chemotherapy in KEYNOTE-689. Additionally, 77.3% (17/22) of patients downstaging of pathological stage, and the treatment improved laryngeal preservation rates. The MPR rate in patients with high PD-L1 expression (CPS ≥ 10) was 54.5%, which was lower than the overall rate. This result may reflect the complexity of the tumor microenvironment, while high PD-L1 expression is generally associated with enhanced sensitivity to immunotherapy, factors, such as an immunosuppressive microenvironment or tumor heterogeneity could limit therapeutic efficacy in this subgroup. Conversely, patients with moderate PD-L1 expression (1 ≤ CPS < 10) exhibited the highest MPR rate (71.4%), suggesting that they derive the greatest benefit from neoadjuvant chemoimmunotherapy. This enhanced response might arise from an optimal balance between immune activation and tumor antigen load **(Figure 5B)**. The stratification by PD-L1 CPS levels revealed significant differences in treatment response, supporting the predictive value of CPS for outcomes with pembrolizumab-based neoadjuvant chemoimmunotherapy. Patients with moderate PD-L1 expression may obtain substantial benefit from this approach, whereas those with high PD-L1 expression might require complementary strategies, such as anti-angiogenic agents or dual ICI, to improve pathological remission rates. These observations are consistent with the KEYNOTE-048 study, which indicated that patients with moderate CPS benefit more from immunotherapy, whereas the CHECKMATE-141 trial found no correlation between CPS values of 5-10 and favorable outcomes. However, a single-center retrospective real-world study from Liaoning Cancer Hospital reported contrasting results. In that study of pembrolizumab combined with neoadjuvant chemotherapy, the overall ORR was 90.5% (19/21), and the patients with CPS ≥ 20 achieved an ORR of 100% (13/13), compared with 75% (6/8) in those with CPS < 20. These results indicate that MPR and ORR are not strictly correlated with CPS, as PD-L1 represents only one immune escape mechanism. Additional pathways, including CTLA-4 and TGF-β, also influence immunotherapy response. In patients with low PD-L1 expression, chemotherapy or immunotherapy may activate anti-tumor immunity via alternative mechanisms, rendering CPS alone insufficient for predicting efficacy. Furthermore, tumor heterogeneity may contribute to discordance between CPS and response to chemoimmunotherapy. For example, immunosuppressive factors such as tumor-associated macrophages (TAMs) or elevated TGF-β expression can diminish the predictive utility of PD-L1. High tumor mutational burden (TMB) can enhance immune recognition by increasing tumor antigen availability, enabling even patients with low PD-L1 expression to benefit from immunotherapy. Finally, combination therapy may attenuate the predictive power of single biomarkers such as CPS, as chemotherapeutic agents can selectively target immunosuppressive cells, (e.g., T cells (Tregs), myeloid-derived suppressor cells (MDSCs), and M2-like TAMs, thereby rebalancing the tumor microenvironment^17^. This microenvironmental remodeling may compensate for low PD-L1 expression in patients with low CPS scores, weakening the correlation between CPS and MPR or ORR.

Pathological staging plays a critical role in determining indications for adjuvant therapy in OSCC/OPSCC. Decisions regarding adjuvant (chemo)radiotherapy are typically based on pretreatment imaging, physical examination, and the pathological staging of surgical specimens. However, in patients who achieve MPR, neoadjuvant immunotherapy may eliminate pathological features that would otherwise indicate adjuvant the need (chemo)radiotherapy, potentially reducing its utilization^18^. In the present study, one of the 25 patients with MPR experienced tumor recurrence at a follow-up of 3.3 months, possibly related to heterogeneous lymph-node response within the MPR group and the potentially limited application of adjuvant therapy in these patients. Moreover, studies have confirmed that despite observable lesion regression after neoadjuvant therapy, residual microscopic lesions and atypical hyperplasia predominantly persist at the primary site. Therefore, reducing the extent of surgery in patients with apparent lesion regression is not recommended. Standardization of safety margins and the intraoperative margin assessment are essential to minimize the risk of residual disease.

In this study, the median OS and EFS were not reached. The results suggests that the most patients remained alive and free of recurrence during the follow-up period, which is closely associated with the substantial benefit of neoadjuvant chemoimmunotherapy in reducing recurrence rate and improving survival. However, the unreached median survival endpoints may also reflect the relatively limited follow-up time, during which most patients have not yet experienced survival events. The current analysis primarily describes early survival trends and subgroup differences, and extended follow-up planned to obtain more mature results. The study demonstrated considerable antitumor activity, with an ORR of 85.4%, a MPR rate of 65.8%, and an R0 resection of 100%. These findings indicate that neoadjuvant chemoimmunotherapy can effectively reduce tumor burden and enhance systemic anti-tumor immunity, thereby lowering the risk of postoperative recurrence. The recurrence rate was only 6%. All recurrences were locoregional (cervical lymph node metastases), with no distant metastasis observed, further confirming the regimen's efficacy in controlling tumor dissemination. Additionally, patient characteristics in this cohort also contributed positively to survival outcomes. All patients exhibited favorable baseline characteristics, including an age range of 39-74 years, an Eastern Cooperative Oncology Group (ECOG) performance status of 0-1, and no distant metastasis at diagnosis. These features indicate a generally favorable baseline health status, enabling better treatment tolerance and potential benefit. Moreover, none of the patients who achieved MPR experienced recurrence during follow-up, suggesting that pathological remission not only reflects the efficacy of preoperative treatment but also serve as an important predictor of long-term survival. Additionally, the synergistic effect of postoperative multimodal therapy further consolidates the benefits of neoadjuvant chemoimmunotherapy Selected patients received adjuvant radiotherapy or chemoradiotherapy, effectively eliminating residual tumor cells, substantially reducing recurrence risk, and prolonging survival. For patients with poor performance status, postoperative single-agent immunotherapy represents a viable alternative, aiming to minimize toxicity while maintaining disease control and improving OS rates. It is noteworthy that the unreached median OS and EFS may be partly attributable to the relatively short follow-up time. The median follow-up time was 31.7 months (95% CI, 29.4-34.0). Although some patients experienced death or recurrence, OS outcomes remained promising. For long-term survival-oriented treatments, longer follow-up is generally required to accurately estimate median OS and EFS. Furthermore, the low event rates for mortality and recurrence (each 6%) limited the number of observed events, which challenges the derivation of reliable statistical estimates for median survival.

Neoadjuvant therapy comprising pembrolizumab, albumin-bound paclitaxel, and cisplatin demonstrated a manageable safety profile in patients with OSCC/OPSCC, without delaying standard therapy. The most common grade ≥ 3 treatment-related adverse events (TRAEs) were anemia (14.0%), alopecia (8.0%), neutropenia (6.0%), thrombocytopenia (6.0%), and pyrexia (4.0%). No treatment-related deaths were observed, although surgery was delayed in 3 (6.0%) patients due to chemotherapy-related toxicity. Additionally, chemotherapy was discontinued in 2 (4.0%) patients due to toxicity. Specifically, one patient could not undergo deep vein catheterization owing to venous thrombosis and switched to immune-targeted therapy, while the other switched to single-agent Kerida treatment after one cycle of chemoimmunotherapy due to bone marrow suppression. Close monitoring for irAEs is recommended during both the neoadjuvant and postoperative periods. The toxicity profile indicates that neoadjuvant chemoimmunotherapy did not result in significant increase in side effects versus conventional chemotherapy, and the incidence of irAEs remained low. For instance, only a small proportion of patients had mild hypothyroidism (10.0%) and pneumonia (10.0%). Compared with other reported immunotherapy regimens, the incidence and severity of irAEs in this study remained within a manageable range^19^.

While this single-center retrospective study provides preliminary evidence and clinical insights supporting the application of this regimen in locally advanced resectable OSCC/OPSCC, several limitations warrant consideration. First, the sample size was small (n=50), and large-scale, controlled studies with extended follow-up are required to validate whether neoadjuvant chemoimmunotherapy can offer sustained survival benefits to the broader OSCC/OPSCC population. Second, owing to the relatively short follow-up duration, the median OS and EFS were not reached. Extended follow-up will yield more comprehensive survival data, particularly given the low rates of recurrence and mortality. Third, the single-arm, retrospective design lacks a randomized control group which potentially introduces selection bias and limits the ability to clearly evaluate the comparative efficacy and safety profile of neoadjuvant chemoimmunotherapy. Specifically, our cohort included both oral and oropharyngeal cancer patients. Despite their anatomical proximity adjacent, there two subsites differ in etiology and biological behavior, a pooled analysis may obscure subgroup-specific variations in therapeutic efficacy. Moreover, the number of neoadjuvant therapy cycles before surgery varied among patients, which may serve as a key confounding factor affecting the accurate assessment of surgical outcomes and adjuvant therapy efficacy. Future studies should address these limitations by prioritizing large-scale, prospective randomized controlled trials (RCTs) with extended follow-up durations. Additionally, personalized treatment strategies for different patient subgroups should be further optimized. These measures will bolster the reliability of the findings and enhance their clinical applicability.

## Figures and Tables

**Table 1:** Baseline characteristics of enrolled participants.

Characteristics	Participants (50)
Median age, year (range)	58 (39-74)
Sex	
Male	38 (76.0%)
Female	12 (24.0%)
Smoking history	
Never	34 (68.0%)
Current or former	16 (32.0%)
Alcohol use	
Never	31 (62.0%)
Current or former	19 (38.0%)
HPV infection	
Positive	6 (46.2%)
Negative	5 (38.5%)
Unknown	2 (15.3%)
Pretreatment clinical T-stage	
T1	2 (4.0%)
T2	15 (30.0%)
T3	14 (28.0%)
T4	19 (38.0%)
Pretreatment clinical N-stage	
N0	22 (44.0%)
N1	16 (32.0%)
N2	10 (20.0%)
N3	2 (4.0%)
PD-L1 CPS	
<1	1 (2.0%)
1-10	10 (20.0%)
≥10	19 (38.0%)
Unknown	20 (40.0%)

**Table 2:** Assessment of short-term efficacy

Short-term efficacy	With MPR n (%)	Without MPR n (%)
Complete remission (CR)	3 (100)	0 (0)
Partial remission (PR)	21 (70)	9(30)
Stable disease (SD)	1(25)	3 (75)
Progressive disease (PD)	0 (0)	1 (100)

Denominator = 3 patients for complete remission (CR); 30 patients for partial remission (PR); 4 patients for stable disease (SD); 1 patient for progressive disease (PD)

**Table 3:** Adverse events potentially related to the treatment.

Chemotherapy-related adverse events	Grade 1	Grade 2	Grade 3	Grade 4	Grade 5
Anemia	10 (20%)	14 (28%)	7 (14%)	0 (0%)	0 (0%)
Neutropenia	6 (12%)	9 (18%)	3 (6%)	0 (0%)	0 (0%)
Thrombocytopenia	5 (10%)	5 (10%)	3 (6%)	0 (0%)	0 (0%)
Alopecia	7 (14%)	38 (76%)	4 (8%)	0 (0%)	0 (0%)
Constipation	16 (32%)	2 (4%)	0 (0%)	0 (0%)	0 (0%)
Diarrhea	6 (12%)	5 (10%)	0 (0%)	0 (0%)	0 (0%)
Nausea	21 (42%)	9 (18%)	2 (4%)	0 (0%)	0 (0%)
Stomatitis	7 (14%)	8 (16%)	0 (0%)	0 (0%)	0 (0%)
Vomiting	10 (20%)	4 (8%)	2 (4%)	0 (0%)	0 (0%)
Fatigue	10 (20%)	4 (8%)	1 (2%)	0 (0%)	0 (0%)
Increased alanine aminotransferase	6 (12%)	5 (10%)	0 (0%)	0 (0%)	0 (0%)
Increased aspartate aminotransferase	5 (10%)	1 (2%)	0 (0%)	0 (0%)	0 (0%)
Increased alkaline phosphatase	11 (22%)	5 (10%)	1 (2%)	0 (0%)	0 (0%)
g-Glutamyltransferase increased	8 (16%)	2 (4%)	0 (0%)	0 (0%)	0 (0%)
Pyrexia	6 (12%)	2 (4%)	2 (4%)	0 (0%)	0 (0%)
Chemotherapy-induced rash	4 (8%)	3 (6%)	0 (0%)	0 (0%)	0 (0%)
**Immune-related adverse events**					
Immune rash	3 (6%)	2 (4%)	0 (0%)	0 (0%)	0 (0%)
Hypothyroidism	5 (10%)	3 (6%)	0 (0%)	0 (0%)	0 (0%)
Hyperthyroidism	1 (2%)	1 (2%)	0 (0%)	0 (0%)	0 (0%)
Immune-related pneumonia	1 (2%)	1 (2%)	0 (0%)	0 (0%)	0 (0%)

Treatment-related adverse events (AEs) were defined as adverse medical events potentially related to the treatment that occurs during the clinical trial. AEs were graded according to the Common Terminology Criteria for AEs (CTCAE), version 5.0, to characterize their severity. The CTCAE severity grades range from 1 to 5, with unique clinical descriptions for each AE, as per the following general guideline: Grade 1: Mild; asymptomatic or mild symptoms; clinical or diagnostic observations only; intervention not indicated. Grade 2: Moderate; minimal, local, or noninvasive intervention indicated; limiting age-appropriate instrumental activities of daily living (ADL). Grade 3: Severe or medically significant but not immediately life-threatening; hospitalization or prolongation of hospitalization indicated; disabling; limiting self-care ADL. Grade 4: Life-threatening consequences; urgent intervention indicated. Grade 5: Death related to AE.
